# Genetic diversity analysis of Morchella sp.by RAPD

**Published:** 2017-03

**Authors:** Muhammad Irfan, Shuang Yang, Luo Yuxin, Jia-Xing Sun

**Affiliations:** 1 Biotechnology Research Laboratory, Shenyang Academy of Agricultural Sciences, Shenyang Liaoning, China.; 2 Department of Biotechnology, University of Sargodha, Sargodha Pakistan

**Keywords:** Morchella Species, PCR-RAPD, Genetic Diversity, Phylogeny

## Abstract

This study investigated the genetic diversity of morchella species using randomly amplified polymorphic DNA markers. Morchella species are an important group of edible mushrooms belonging to the family Helveliaceae with medicinal and economical significance. In this study we have developed an efficient method of genomic DNA isolation which was amplified by eight RAPD primers to test the polymorphism in three species of morchella. Out of all eight primers tested in current study, one of them (B8) resulted 100% polymorphism among the three studied species. Based on the RAPD profile a similarity matrix was generated to construct a dendrogram for phylogenetic analysis revealing the relationship among the three species of morchella.

## INTRODUCTION

Morels belong to the family Helveliaceae of fungi which are an important group of mushrooms due to its edible value. The fruit bodies of all the Morchella species are edible and are mainly used as flavoring in soups and gravies. Morels are cylindrical in shape consisting of upper part cap and a lower part stalk and are polymorphic. The upper part (cap) of a morel is called pileus and contains 70 to 80% of the total weight of the fungus. The pileus is generally brown, yellow, black or pale in color. The stalk of morel is 1.0 to 4.0 cm long, 0.5 to 3.0 cm thick, hollow and of variable shapes [1]. The polymorphism in morels is due to head shape, stalk-to-head ratio, color (immature and mature), taste and edibility [2,3]. Researchers have reported about three to fifty species of morchella species [2]. The metabolites produced by morels are clinically used as an anti-tumor agent [4]. 

Presence of high polysaccharide contaminants, tough cell walls and high level of intracellular ribonuclease create difficulties in isolating high quality DNA from fungi [5,6]. Various protocols have been established for fungal DNA which is not efficient in all fungi and are specific to particular fungal species [6]. Extraction of genomic DNA from fungi involves two main steps, breaking of cell wall and extraction purification of genomic DNA. Mostly CTAB is used for extraction buffer [7] and purification is done by phenol /chloroform extraction and isopropanol or ethanol precipitation [8]. Major problem is the breakdown of cell wall of the fungus which was done by using various methods like liquid nitrogen or glass rods [9], dry ice [10], enzyme digestion [11], glass or magnetic beads [12], benzyl chloride [13], microwave exposure [14] and combinations of different methods [15].

Various molecular techniques have been developed to classify the morel systematics. Among these techniques starch gel electrophoresis [16], enzyme-linked immunosorbent assay [17], restriction-length polymorphism (RFLP) analysis of 28S and 18S ribosomal RNA genes [5] and sequences of internal transcribed spacer (ITS) region of genomic DNA have been done phylogenetic analysis of *Morchella *species [18]. RAPD markers were mostly used to check the genetic similarity and phylogenetic analysis due to the ease in its methodology [19]. In most cases, the individuals look similar in morphology but they are genetically different. So in this study, an attempt was made to use RAPD analysis to check the genetic diversity analysis in morel species.

## MATERIALS AND METHODS

**Cultivation of Fungus: **All three species of Morchella, which -were obtained from Shenyang academy of agricultural sciences, were cultivated in potato dextrose broth at 30^o^C with agitation speed of 150 rpm. The fungi were developed into ball like structures, then they were used for DNA extraction.

**DNA Extraction and RAPD-PCR reaction: **The DNA of the Morels were extracted following two different methods; (a) SDS method: Approximately 0.1g of dried morel was taken in eppendorf tube and ground to powder form by liquid nitrogen. After that 0.5 ml of 0.5% SDS was added and centrifuged at 12000 x g for 2 min. Next, 150 µl of supernatant was transferred to another eppendorf tube and 150 µl of isopropanol was added in it and centrifuged at 12000 x g for 2 min. Finally, the supernatant was discarded, pellet was dried and 100 µl of sterilized distilled water was added in each tube and checked on 0.8% agarose gel. (b) Kit method: Kit was supplied by SBS Genetech Co. Ltd. China which is commonly known to be used for DNA extraction from plant. Here we have applied this kit to extract the DNA from fungus with some modification as per protocol provided by the company. 

RAPD-PCR reaction was performed by using 8 primers listed in Table 1, which were obtained from SBS Genetech Co. Ltd. China. RAPD-PCR reaction was performed following the method described by Williams et al [20]. Each PCR reaction consisted of 5µl mix (Taq Polymerase + dNTPs), 2 µl of 2.5mM primer, 1µl DNA, 2µl dd water with total volume of 10 µl. Amplifications were carried out in a thermocycler (Eppendorf) with following protocol: 5 min of denaturation at 94°C followed by 40 cycles of 40 seconds at 94°C, 2 min at 37°C, 45 seconds at 72°C followed by 5 min final extension at 72°C. The RAPD products after PCR were separated by electrophoresis on 0.8% agarose gel in 1X TBE (Tris boric acid EDTA) buffer using ethedium bromide staining and visualized under UV light.

**Table 1:** List of primers used during this study

**O** **ligo name**	**Sequence**	**O** **ligo name**	**Sequence**
B3	CATCCCCCTG	B7	GGTGACGCAG
B4	TGCGCCCTTC	B8	GTCCACACGG
B5	TGCGCCCTGC	B9	TGGGGGACTC
B6	TGCTCTGCCC	B10	CTGCTGGGAC

**Data analysis: **Data was analyzed by comparing the RAPD profile on the basis of the presence or absence (1 or 0 respectively) of each score able DNA bands. Each scorable band was considered as single locus/allele. A similarity matrix was generated using Nei and Li’s [21] coefficient of similarity and a dendrogram was generated using SHAN function of NTSYSpc software version 1.80 [22].

## RESULTS AND DISCUSSION

In this study, DNA extraction method was optimized and genetic diversity of morchilla sp was done by RAPD primers. DNA extracted by SDS method had no producible results then kit method was employed for DNA extraction. In normal kit method protocol, 15 minute of incubation was mentioned for DNA extraction from plants which was used for morels DNA extraction. Here again no proper results were found. Then we have changed the incubation time to 60 minutes with interval of 15 minutes. Results showed that 60 minute of incubation time gave better results as compared to 15, 30 and 45 minutes respectively. 

**Figure 1:**
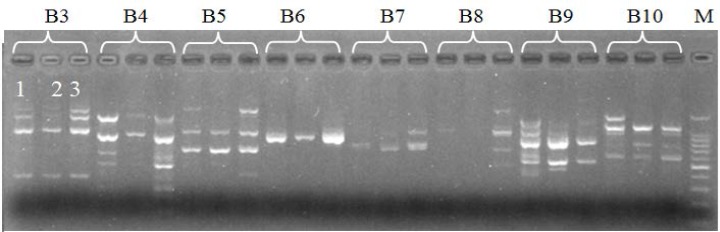
RAPD profile of the fungus Morchilla sp. Lanes 1, 2, and 3 represents different sepcies of Morchella.

Amplification products were ranged from 100bp to 1100bp in size, using standard DNA marker (Fig. 1). The number of amplification products produced by each primer was 3 (B6) to 9 (B3, B4, B9) with average of 6.625. Total of 53 scorable fragments were produced by eight RAPD primers from three species of Morels. Among these 53 fragments, 18 fragments were found to be polymorphic ranging from 2 to 6 fragments with average of 2.63 fragments per primer. Among these eight primers, B8 showed 100 % polymorphism while B6 showed no polymorphism. The primer B4 yielded highest number (6) of polymorphic bands with polymorphism of 66.6% while the primers B5 and B10 yielded 2 polymorphic fragments representing 25% polymorphism (Table 2). Each primer produced different numbers of bands which might be due to the difference in sequences, availability of annealing sites in the genome and quality of the template [23]. Fazal et al [1] collected five different species of Morels which were amplified by four RAPD primers indicating fragment size of 100 to 1100bp.

**Table 2:** Number of alleles detected and polymorphism produced by eight RAPD primers

**Primers**	**Observed number of alleles**		**Polymorphic alleles**	**Polymorphism (%)**	
**B3**	9	3		33.3
**B4**	9	6		66.6
**B5**	8	2		25.0
**B6**	3	0		00.0
**B7**	4	2		50.0
**B8**	3	3		100
**B9**	9	3		33.3
**B10**	8	2		25.0
**Mean**	6.625	2.625		
**Total**	53	18		

**Figure 2:**
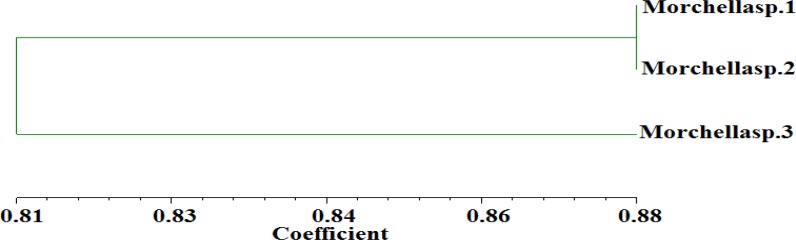
Dendogram of three Morchella sp based on 8 RAPD primers

On the basis of this RAPD analysis, a dendrogram was constructed (Fig. 2) From this result it was found that these three morchella species was clustered into two groups. Morchella sp. one and two had same group while morchella specie three exhibit another group. So, from this analysis it was concluded that morchella specie 3 is different from other two species, but all the three species were closely related to each other. In another study Dalgleish and Jacobson [24] used RAPD-PCR indicating high genetic variations among *M. esculenta* populations collected from three different sites in the United States. Various studies reported that random amplified polymorphic DNA (RAPD) was widely used for the estimation of genetic diversity in mushrooms [25].
